# High-Level Heteroatom Doped Two-Dimensional Carbon Architectures for Highly Efficient Lithium-Ion Storage

**DOI:** 10.3389/fchem.2018.00097

**Published:** 2018-04-05

**Authors:** Zhijie Wang, Yanyan Wang, Wenhui Wang, Xiaoliang Yu, Wei Lv, Bin Xiang, Yan-Bing He

**Affiliations:** ^1^CAS Key Lab of Materials for Energy Conversion, Department of Materials Science and Engineering, Synergetic Innovation Center of Quantum Information Quantum Physics, University of Science and Technology of China, Hefei, China; ^2^Engineering Laboratory for the Next Generation Power and Energy Storage Batteries, Engineering Laboratory for Functionalized Carbon Materials, Graduate School at Shenzhen, Tsinghua University, Shenzhen, China; ^3^China Key Laboratory of Optoelectronic Devices and Systems of Ministry of Education and Guangdong Province, College of Optoelectronic Engineering, Shenzhen University, Shenzhen, China; ^4^Center for Green Research on Energy and Environment Materials, National Institute for Materials Science, Tsukaba, Japan

**Keywords:** 2D carbon nanomaterials, hierarchical structure, high-level heteroatom doping, Li-ion batteries, high-rate capability

## Abstract

In this work, high-level heteroatom doped two-dimensional hierarchical carbon architectures (H-2D-HCA) are developed for highly efficient Li-ion storage applications. The achieved H-2D-HCA possesses a hierarchical 2D morphology consisting of tiny carbon nanosheets vertically grown on carbon nanoplates and containing a hierarchical porosity with multiscale pore size. More importantly, the H-2D-HCA shows abundant heteroatom functionality, with sulfur (S) doping of 0.9% and nitrogen (N) doping of as high as 15.5%, in which the electrochemically active N accounts for 84% of total N heteroatoms. In addition, the H-2D-HCA also has an expanded interlayer distance of 0.368 nm. When used as lithium-ion battery anodes, it shows excellent Li-ion storage performance. Even at a high current density of 5 A g^−1^, it still delivers a high discharge capacity of 329 mA h g^−1^ after 1,000 cycles. First principle calculations verifies that such unique microstructure characteristics and high-level heteroatom doping nature can enhance Li adsorption stability, electronic conductivity and Li diffusion mobility of carbon nanomaterials. Therefore, the H-2D-HCA could be promising candidates for next-generation LIB anodes.

## Introduction

Lithium ion batteries (LIBs) have been regarded the most important power sources for portable electronic devices and promising candidates to power future electric vehicles (Armand and Tarascon, 2008; Geng et al., 2018). In order to meet the increasing demand for energy density and fast discharge-charge abilities, it is urgent to develop LIB electrode materials with higher specific capacities, better rate capabilities and excellent cycle stabilities (Arico et al., 2005; Zhang et al., 2018). Graphite has served as the most popular anode materials for its low price, appropriate working voltage platform and high Columbic efficiency (Yazami and Touzain, 1983; Kaskhedikar and Maier, 2009; Lu et al., 2017). Unfortunately, it suffers from limited Li storage capacity (372 mA h g^−1^, according to the intercalation mechanism with the formation of LiC_6_ composites) and poor rate performance, which cannot satisfy the practical application requirements (Yazami and Touzain, 1983; Lu et al., 2017). Therefore, developing advanced alternative materials to replace graphite has attracted great research interest in recent years (Kaskhedikar and Maier, 2009; Zhang Q. et al., 2016; Wu et al., 2017; Deng et al., 2018).

Current researches have already proved that carbon nanomaterials delivered better LIB performance than graphite, since abundant Li-ion storage sites and rapid ion diffusion channels can be provided (Zhou et al., 2003; Dai et al., 2012; Zhang Q. et al., 2016; Wang et al., 2018). In addition, successful structural modification could further enhance the electrochemical performance of nanocarbons (Wu et al., 2003; Landi et al., 2009). Heteroatom doping plays an important role in the modification because it can adjust the physical and chemical properties of carbon nanomaterials (Wu et al., 2011). For instance, both experimental and theoretical results demonstrated that nitrogen (N) doping can positively affect the electric conductivity and electrochemical activity of nanocarbons (Ma et al., 2012; Zheng et al., 2014). Especially pyrrolic N (N-5) and pyridinic N (N-6) are able to create active sites for Li-ion adsorption in the carbon framework. Hence, increasing the doping concentration of N-5 and N-6 is beneficial to the LIB performance of nanocarbon electrodes (Wang et al., 2011; Ma et al., 2012; Mao et al., 2012; Zheng et al., 2014). Besides, sulfur (S) heteroatoms can enlarge the interlayer distance of carbons because of the larger covalent radius (102 pm) compared with that of C (77 pm) (Qie et al., 2015; Xu et al., 2016). The enlarged interlayer distance facilitates the insertion-extraction of electrolyte ions, and thus is able to improve rate capabilities (Qie et al., 2015; Xu et al., 2016; Liang et al., 2018). It is worth noting that, Li-ion storage performance of carbon nanomaterials can be promoted not only by improving heteroatom doping concentration, but also by the synergistic effects between different kinds of dopants (Ai et al., 2014). Therefore, high-level N, S co-doping can be an effective strategy to achieve high-performance nanocarbon anodes.

In the previous work, we constructed two-dimensional (2D) hierarchical carbon architectures (2D-HCA) with N, S co-doping nature for superior LIB anodes (Wang et al., 2016). Here, we further developed high-level heteroatom doped 2D hierarchical carbon architectures (H-2D-HCA) with enhanced Li-ion storage performance by increasing the N concentration in 2D-HCA. The obtained H-2D-HCA contains a much higher heteroatom concentration of 16.4%, with 15.5% of N and 0.9% of S. Interestingly, the electrochemically active N, i.e., N-5 and N-6, accounts for the majority of total N atoms (47 and 37%, respectively). Furthermore, it has an expanded interlayer space of 0.368 nm compared with that of graphite, which can enhance the Li ion diffusion speed. Benefiting from such high doping level and the unique microstructure, the H-2D-HCA can be used for highly efficient Li-ion storage. To be specific, even at a high current density of 5 A g^−1^, it still delivered a high specific capacity of 329 mA h g^−1^ after 1,000 cycles.

## Experimental methods

### Preparation of H-2D-HCA

Mg-Al layered double hydroxides (Mg-Al LDH) and Mg-Al layered double oxides (Mg-Al LDO) were prepared following our previous work (Wang et al., 2016). Typically, Mg(NO_3_)_2_·6H_2_O (12.82 g, 99.9%, Macklin), Al(NO_3_)_2_·9H_2_O (9.38 g, 99.9%, Macklin) and urea (90.09 g, 99.9%, Macklin) were dissolved into 500 ml water in a round-bottom flask. Then the solution was heated 100°C and kept for 12 h under magnetic stirring. After that, the temperature was decreased to 94°C and kept for another 12 h without stirring. The produced white powder was collected with vacuum filtration and freeze dried, then Mg-Al LDH was obtained. As-prepared Mg-Al LDH was calcined at 500°C for 3 h in a muffle furnace and ground to get Mg-Al LDO. To adsorption OII, 150 ml water was filled into a three-mouth-flask. After driving the dissolved air with Ar gas flow, 0.6 g Orange II (>85%, Macklin) was dispersed into the DI water. Then, 1 g Mg-Al LDO was added to the solution to adsorb the dye for 48 h. Magnetic stirring and Ar gas flow were maintained during the overall adsorption process. Afterwards, the solution was vacuum filtrated and washed with water for several times, then the obtained powder (RLDH/OII) was freeze dried and ground. The obtained RLDH/OII was mixed with 0.6 g melamine (99%, Macklin), and the mixture was dispersed into 50 mL methanol in a beaker. After that, the solution was heated to 50°C under magnetic stirring. The temperature was kept until the methanol was totally evaporated, and then the RLDH-OII-melamine mixture (RLDH-OII-M) was collected. The carbonization of RLDH-OII-M was performed at 800°C for 2 h under Ar atmosphere with a heating rate of 2°C/min. Then the obtained black powder was washed with HCl (6M) and NaOH (2M) respectively at 70°C for several hours. After freeze drying process, the target product H-2D-HCA was obtained.

2D-HCA was synthesized as reference samples followed the above procedures except melamine mixing process. OII and OII-melamine mixture (mass ratio: 1:1) were carbonized and washed with the above-mentioned conditions to get the reference sample C-OII and high-level doped C-OII (H-C-OII), respectively.

### Microstructure characterization

The morphology observation and elemental mapping analyzing were conducted by using a field electron microscopy (FESEM, ZEISS SUPRA55). The microstructures of the four samples were examined by a field high resolution transmission electron microscopy (HRTEM, FEI Tecnai G^2^ F30). A Rigaku D/max 2500/PC diffractometer (Cu-Kα radiation with λ = 1.5418 Å) was used to measure the powder X-ray diffraction (XRD) patterns of samples. A HORIBA Labram HR Evolution Raman spectrometer was used to measure the Raman spectra of the samples (the excitation wavelength of the leaser is 532 nm). The XPS analyses were carried out on a PHI 5000 VersaProbe II spectrometer using monochromatic Al K(alpha) X-ray source. The N2 adsorption/desorption isotherms were measured by using a Micromeritics ASAP 2020 automated adsorption apparatus at 77 K. The specific surface areas was determined based on Brunauer-Emmett-Teller (BET) equation, and the pore-size distribution was calculated by utilizing density functional theory (DFT).

### Electrochemical tests

Li-ion storage performance of these samples was tested with half-battery methods by using coin-type cells (CR2032). To prepare the working electrodes, a slurry consisted a mixture of 70 wt % of H-2D-HCA (or other three carbon samples), 20 wt % of poly (vinylidene fluoride) (PVDF) and 10 wt % of acetylene black in N-methyl pyrrolidone was coated on a copper foil. After being dried at 80°C for 12 h, the electrode was punched into disks with a diameter of 12 mm. The mass loading of active materials was ~ 1.2 mg cm^−2^. 1.0 M LiPF_6_ dissolved in ethylene carbonate/diethyl carbonate (EC/DEC, with a volume ratio of 1:1) was used as the electrolyte. Lithium foil was used as both the counter electrode and reference electrodes. Battery assembling was carried out in an Ar-filled glove box with both moisture and oxygen concentrations below 1 ppm. Cyclic voltammetry was measured using an electrochemical workstation (CHI 660E) in the potential window of 0.005–3 V vs. Li/Li^+^ with a scanning rate is 0.2 mV s^−1^. The cycling performance and rate capabilities were tested at in the potential window of 0.005–3 V vs. Li/Li^+^ using a LAND battery tester (CT2001A).

## Results and discussion

**Figure 1:**
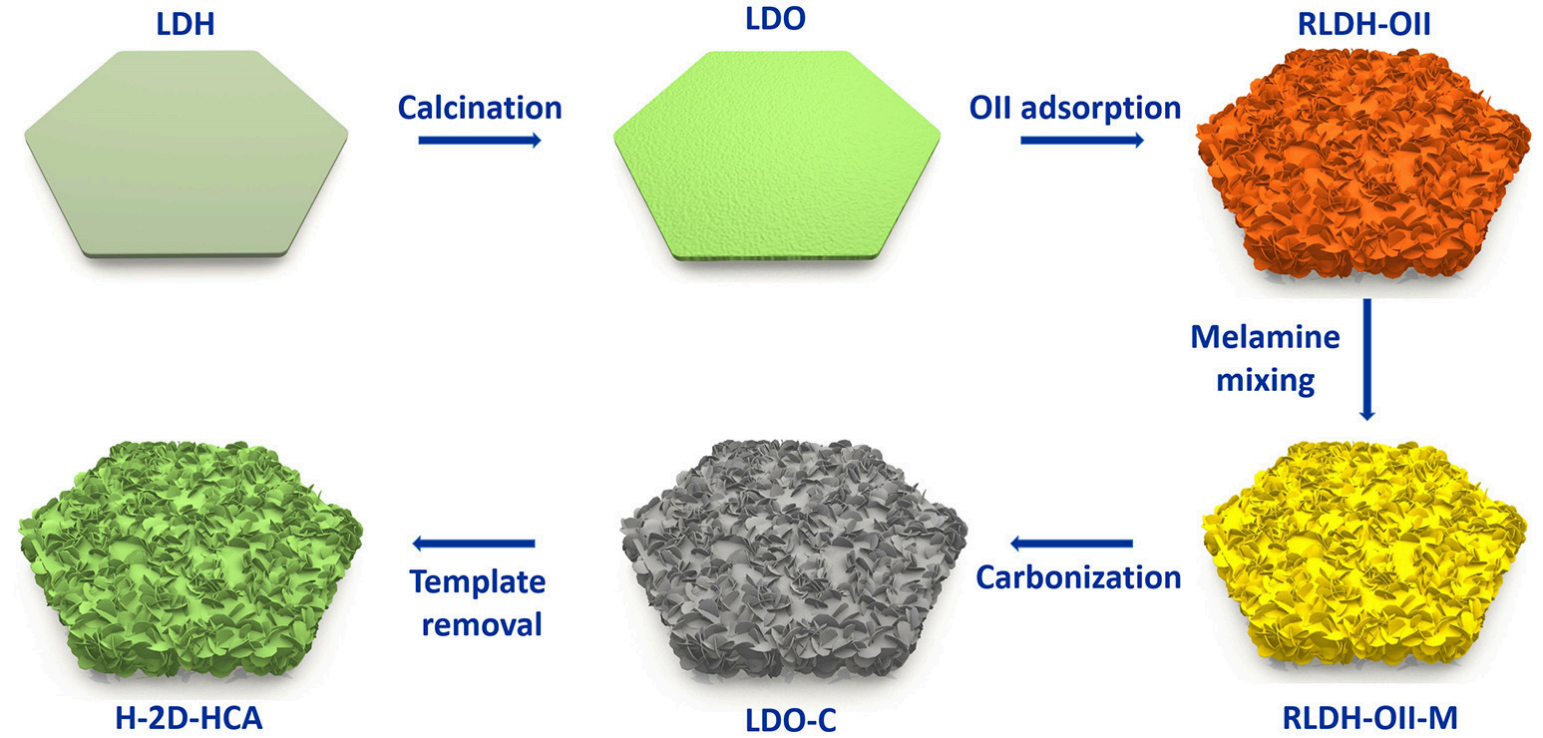
Synthesis approach of H-2D-HCA.

H-2D-HCA was synthesized based on a template-assistant method that we reported before (Wang et al., 2016). Mg-Al layered double oxides (Mg-Al LDO, LDO for short) were used as the templates. Orange II (OII for short, an N- and S-containing organic dye) was used as both carbon precursors and heteroatom sources, and melamine were used as extra N sources. The preparation approach is illustrated in Figure 1 Firstly, Mg-Al layered double hydroxides (Mg-Al LDH, LDH for short) were calcined to derive LDO. Afterwards, the LDO was dispersed into OII solution to adsorb this organic dye. During this process, the LDO was rehydrated to LDH (RLDH). More importantly, a morphology change has occurred simultaneously. This should be attributed to the morphology change occurred in the rehydration process of LDO (Wang et al., 2016). As a result, the obtained RLDH-OII composite had a 2D hierarchical structure, rather than a 2D smooth plating structure like LDH or LDO. After that, the RLDH-OII was mixed with melamine in methanol under magnetic stirring, and a RLH-OII-melamine mixture (RLDH-OII-M) was collected. Then, the RLDH-OII-M was heated at 800°C to carbonize the organic, meanwhile, RLDH was calcined to LDO again. Finally, the obtained LDO-carbon (LDO-C) mixture was washed with NaOH and HCl to remove the LDO templates, and H-2D-HCA was achieved at last. Detailed procedures can be seen in the Experimental Methods section.

**Figure 2:**
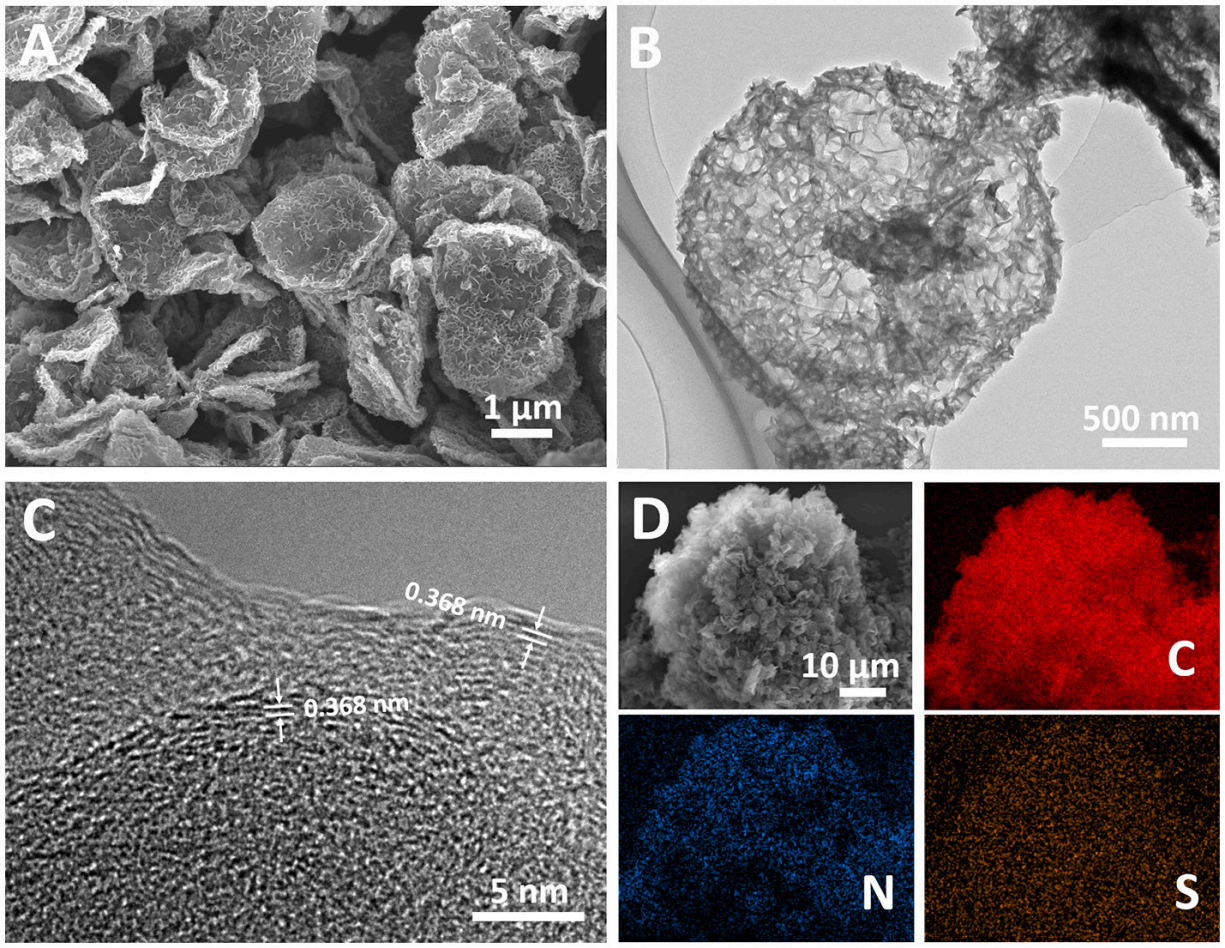
**(A)** Scanning electron microscopy image of H-2D-HCA; **(B)** Transmission electron microscopy image of H-2D-HCA; **(C)** HRTEM image of H-2D-HCA; **(D)** SEM elemental mapping of H-2D-HCA.

Both LDH and LDO have a hexagonal plating morphology with a smooth surface (Supplementary Figure 1). However, after OII adsorption and melamine mixing process, RLDH-OII and RLDH-OII-M show a hierarchical structure of some small-size sheets decorating on the surface of the hexagonal plates (Supplementary Figure 2). This should be attributed to the morphology change during the rehydration process of LDO (Wang et al., 2016). After carbonization and template removal process, the obtained H-2D-HCA has successfully maintained this kind of hierarchical structure, in which small carbon nanosheets growing on the surface of large-size hexagonal carbon nanoplates (Figure 2A). The diameter of H-2D-HCA is ~2.2 μm (Figure 2B) and the average thickness is ~200 nm (Supplementary Figure 3). The small carbon nanosheets growing on the surface of H-2D-HCA have a size of ~100 nm and a thickness of ~10 nm (Supplementary Figure 3). The adjacent carbon nanosheets have formed many half-open pores with diameters of 10 of nanometers (Supplementary Figures 3, 4). These pores can act as reservoirs to storage electrolyte and guarantee good contact of the nanocarbon electrodes with electrolyte. 2D-HCA has similar hierarchical structure and the size with H-2D-HCA (Supplementary Figure 5), which was also reported in the previous work (Wang et al., 2016). This kind of hierarchical structure can alleviate aggregation issues of carbon nanomaterials, which caused by π-π interaction between carbon layers, and thus decrease the electrochemically active surface loss (Zhao et al., 2014; Yu et al., 2015). For comparison, H-C-OII and C-OII have irregular structure with size of about tens of micrometers (Supplementary Figure 5). These structures do not benefit electrolyte access to carbon electrodes and hinder the diffusion of Li ions.

The microstructure of H-2D-HCA was investigated by high-resolution transmission electron microscopy (HRTEM). Interestingly, H-2D-HCA has an amorphous nature and graphitic micro-crystallites with an average interlayer distance of 0.368 nm (Figure 2C), which is larger than that of graphite (Ou et al., 2017; Yang et al., 2018). The broaden peaks in the X-ray diffraction (XRD) pattern also confirms the amorphous nature of H-2D-HCA (Supplementary Figure 6). The peak located at 24.2° can be assigned to (002) diffraction peak of graphite (Xu F. et al., 2015; Zheng et al., 2015; Zhao et al., 2016; Yang et al., 2017). This peak corresponds to an interlayer distance of 0.367 nm (calculated based on the Bragg's law), which agrees well with the HRTEM results. The enlarged interlayer distance can promote Li-ion diffusion and help to improve the high rate performance (Qie et al., 2015; Xu et al., 2016; Liang et al., 2018). 2D-HCA has a similar amorphous structure with few graphitic layers, while H-C-OII and C-OII only show amorphous porous structure without graphite micro-crystallites could be observed (Supplementary Figure 7).

Figure 2D shows SEM elemental mapping. It revealed that H-2D-HCA contained not only C elements, but also abundant N and S heteroatoms. The doped N and S distribute uniformly throughout the carbon framework.

**Figure 3:**
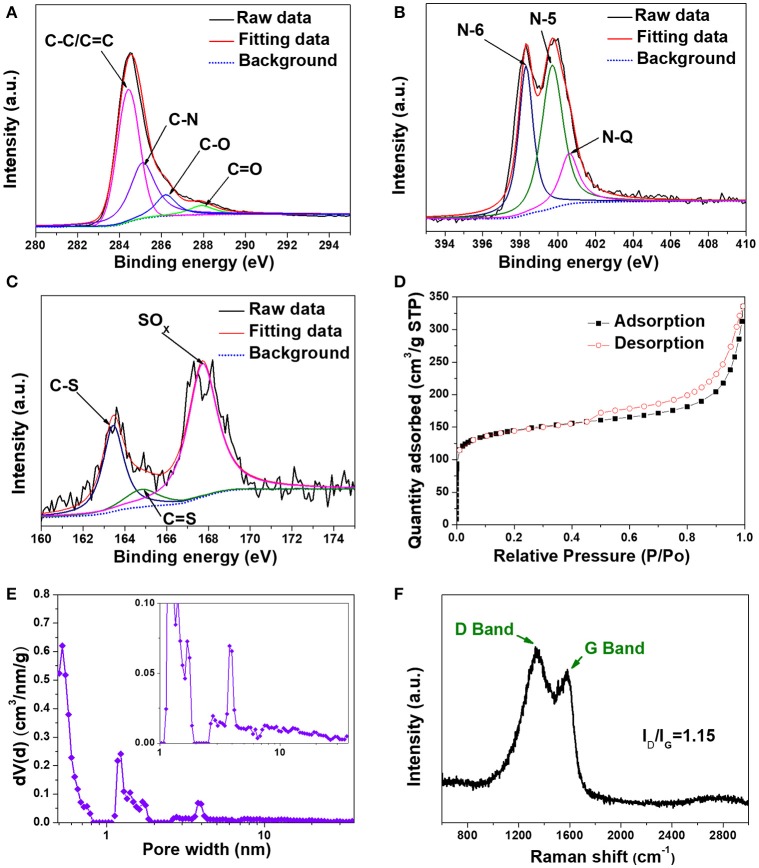
Structural characterization of H-2D-HCA. **(A)** High-resolution XPS spectrum of C1s; **(B)** High-resolution XPS spectrum of N1s; **(C)** High-resolution XPS spectrum of S2p; **(D)** N2 adsorption/desorption isotherms; **(E)** Pore-size distribution curve and inset: enlarged pore-size distribution; **(F)** Raman spectrum.

X-ray photoelectron spectroscopy (XPS) was utilized to evaluate the surface chemistry in H-2D-HCA. N and S heteroatom concentrations can be determined to be 15.5 and 0.9%, respectively (Supplementary Figure 8). The total N and S heteroatom doping level of 16.4% in H-2D-HCA, which is much higher than that of most N, S dual-doped carbon nanomaterials (Supplementary Table 1) (Ai et al., 2014; Sun et al., 2015; Xu G. et al., 2015; Zhou et al., 2015; Zhuang et al., 2015; Shan et al., 2016; Zhang et al., 2016a). The high-resolution spectrum of C1s can be fitted to four peaks (Figure 3A). The peak located at 284.4 eV can be attributed to C-C/C = C of carbon; and the peak located at 285.1 eV reveal the presence of C-N and C-S (Xu G. et al., 2015; Zhuang et al., 2015; Zhang et al., 2016a). The peaks located at 286.2 and 288 eV can be assigned to C-O and C = O, respectively. The high-resolution N1s spectrum can be deconvoluted into three different peaks located at 398.3, 300.8, and 401.1 eV, corresponding to pyridinic-N (N-6), pyrrolic-N (N-5) and graphitic-N (N-Q), respectively (Figure 3B; Wang et al., 2011; Ma et al., 2012; Mao et al., 2012; Zheng et al., 2014; Li et al., 2017). N-6, N-5, and N-Q accounted for 37, 47, and 16% of total N atoms, respectively (Table 1). The electrochemically active N species, N-6 and N-5, occupied a large proportion of total N atoms (84%). The fine split peaks in high-resolution of S2p spectrum indicated the presence of C-S (163.5 eV), C = S (164.8 eV) and SO_X_ group (167.7 eV) (Figure 3C; Zhang X. et al., 2016). As demonstrated by other work, S heteroatom can enlarge the interlayer distance of carbon nanomaterials because of the larger covalent radius (Qie et al., 2015; Xu et al., 2016). The extended interlayer space can promote insertion-extraction speed of Li ions in carbon nanomaterials and thus was expected to improve the fast charge-discharge properties. The elemental composition of H-2D-HCA, 2D-HCA, H-C-OII, and C-OII are summarized in Table 1 (the data of H-C-OII and C-OII were summarized based on XPS results shown in Supplementary Figures 9, 10, and the data of 2D-HCA were calculated from the Reference of Wang et al., 2016). Obviously, H-2D-HCA has the highest doping level and electrochemically active N proportion. Besides, because of the higher N concentration, H-2D-HCA has a much higher heteroatom doping level than 2D-HCA, and H-C-OII also has a much higher heteroatom doping level than C-OII (Table 1). This should be attributed to the use of melamine as extra N source during the synthesizing approach (Sheng et al., 2011).

**Table 1:** Surface physiochemical properties of various samples by XPS tests.

**Sample**	**Elemental composition (%)**				**% of total N atoms**			
	**C**	**N**	**S**	**O**	**N-5**	**N-6**	**N-Q**	**N-5 & N-6**
H-2D-HCA	73.8	15.5	0.9	9.8	47	37	16	84
2D-HCA	86.1	2	2.1	9.8	31.7	20.7	47.6	52.4
H-C-OII	72.7	12.8	0.5	14	37	28	35	65
C-OII	86.6	2.9	1.5	8.3	47.1	36	16.9	83.1

The N_2_ adsorption/desorption isotherms of H-2D-HCA exhibit a combination of type I and type II characteristics, with a distinct hysteresis lop at relative pressure P/P0 ranging from 0.42 to 1 (Figure 3D). The specific surface area (SSA) can be calculated to be 535 m^2^ g^−1^, which is lower than that of 2D-HCA (Wang et al., 2016). The lower SSA could be attributed to melamine decomposition during carbonization process, and this phenomenon was also reported by other previous work (Zheng et al., 2011). Pore-size distribution (PSD) curves reveal that the H-2D-HCA had both micropores which peaks located at 0.52, 1.23, and 1.71 nm, and mesopores which peaks located at 3.97 nm (Figure 3E). The presences of mesopores with size ranging from 6 to 30 nm can also be seen in the enlarged PSD curve (inset of Figure 3E). The co-existence of micropores and mesopores in H-2D-HCA suggests its hierarchical porous structure (Wang et al., 2008; Hu et al., 2015). The hierarchical porous structure benefits LIB performance of nanocarbon electrodes, in which the micropores offer abundant active sites for Li-ion storage and the mesopores can promote the rapid diffusion of Li ions (Zhao et al., 2012). By comparison, the pores in H-C-OII (Supplementary Figure 11) and C-OII (Wang et al., 2016) are mainly micropores (size less than 2 nm), with the much less presence of mesopores.

Figure 3F shows the Raman spectrum of H-2D-HCA. Two remarkable peaks of D band located at 1,341 cm^−1^ and G band at 1,581 cm^−1^ can be observed. As is known, G band corresponds to the zone center E2g mode related to phonon vibrations in sp2 carbon materials, and D band related to structural defects (such as edge, heteroatoms, etc.) and partially disordered structures of the sp2 domains framework (Ferrari et al., 2006). The high I_D_/I_G_ ratio of 1.15 for H-2D-HCA and 1.11 for H-C-OII (Supplementary Figure 12) indicated that they have low graphitization degree and abundant defects (Ferrari et al., 2006). The defects mainly include the heteroatoms doped in the carbon framework. In contrast, because of the lower heteroatom doping level, 2D-HCA and C-OII have lower I_D_/I_G_ ratio of 0.96 and 0.98, respectively (Supplementary Figure 12).

**Figure 4:**
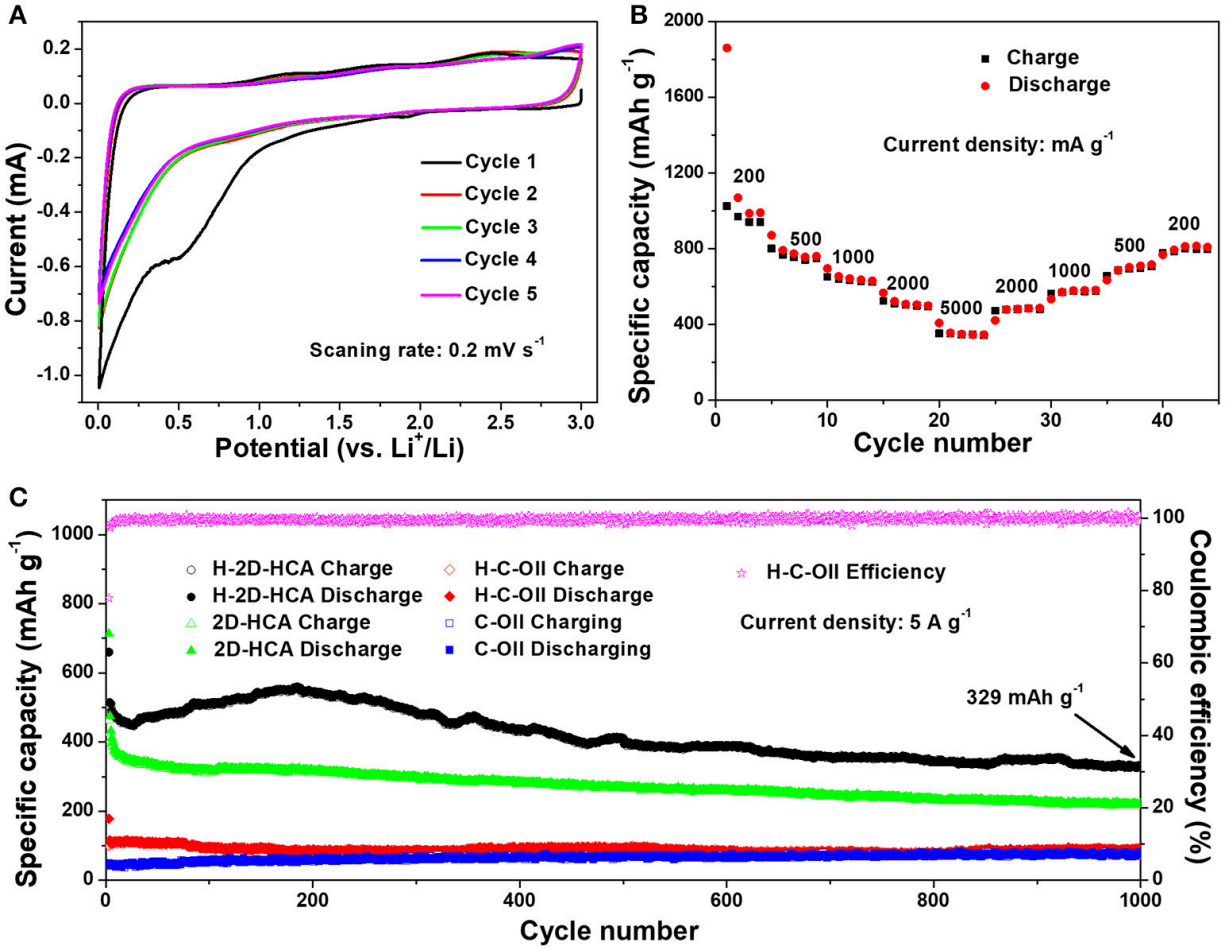
CV curves of H-2D-HCA for the first five cycles, and the canning rate is 0.2 mV s^−1^; **(B)** Rate performance of H-2D-HCA at different current densities; **(C)** Long-term cycling performance of H-2D-HCA, 2D-HCA, H-C-OII, and C-OII at a current density of 5 A g^−1^ along with the corresponding coulombic efficiency of H-2D-HCA.

Li-ion storage properties of H-2D-HCA were evaluated using half-battery test methods. Cyclic voltammetry (CV) curves suggest that H-2D-HCA has similar Li-ion storage behaviors with other carbon nanomaterials. As shown in Figure 4A, in the first cycle, the pronounced irreversible anodic peak at around 0.5 V relates to the electrochemical decomposition of electrolyte and the formation of solid electrolyte interface (SEI) on the huge surface (Sun et al., 2015; Xu G. et al., 2015; Deng et al., 2016; Zhang et al., 2016b). The followed anodic peak near 0 V corresponds to the electrochemical intercalation of Li ions into graphitic structures. The cathodic peaks at 0.2 and 1.2 V can be assigned to Li ions extraction from graphitic layers and defect sites, respectively (Zhang et al., 2016b). The curves in the following cycles overlap together, suggesting the stable formation of SEI layers. When applied as anodes for LIBs, H-2D-HCA exhibits high specific capacity and excellent rate performance. As shown in Figure 4B, H-2D-HCA delivers an initial discharge capacity of 1,861 mA h g^−1^ at current density of 200 mA g^−1^. When the current density increases to 500, 1,000, 2,000, 5,000 mA g^−1^, the discharge capacities of H-2D-HCA were 756, 636, 504, and 348 mA h g^−1^, respectively (measured from the middle cycle in each current density). After that, with the current density returns to 2,000, 1,000, 500, and 200 mA g^−1^, the discharge capacity recovers to the initial capacity values with only little fade.

Long-term cyclability at high current density has been tested to further examine Li-ion storage performance of H-2D-HCA. For comparison, 2D-HCA, H-C-OII and C-OII have been tested at the same condition. First, all the samples were firstly activated at a low current density of 200 mA g^−1^ for two cycles, and then the current density was directly increased to 5 A g^−1^. The voltage profiles of these samples in the first two cycles are shown in Supplementary Figure 13. H-2D-HCA delivered an initial discharge capacity and charge capacity of 1,924 and 1,824 mA h g^−1^, together with an initial columbic efficiency (ICE) of 56.4% (Figure 4C). Because of the large surface area and porous structure, the H-2D-HCA delivered much higher specific capacity than graphite. The irreversible capacity loss in the first cycle is due to the SEI formation and the irreversible insertion of Li ions into micropores. H-2D-HCA has the highest ICE in these four samples (Supplementary Table 2). However, it was still far from satisfaction for practical application. Reducing surface area, coating with dense carbon layer, optimizing pore structure and pre-lithiation may have positive effects on improving the ICE. In addition, it is noted that the ICE of H-2D-HCA is higher than that of 2D-HCA, and the ICE of H-C-OII is higher than that of C-OII, suggesting that increasing N doping level may improve the ICE of nanocarbon anodes. When the current density increased to 5 A g^−1^, H-2D-HCA delivers a discharge capacity of 660 mA h g^−1^ and a CE of 77.9%. The CE rises to a value of higher than 97% in the followed cycles (Figure 4C). Then the discharge capacity of H-2D-HCA drops to 450 mA h g^−1^ in 25 cycles, and increases afterwards, reaches 550 mA h g^−1^ in the 196th cycles. After that, the discharge capacity remains stable only with slight decay. Even after 1,000 cycles, H-2D-HCA can still deliver a high discharge capacity of 329 mA h g^−1^, and a high CE of 99.2% (Figure 4C). In contrast, 2D-HCA delivered a first discharge capacity of 713 mA h g^−1^ and a corresponding CE of 65% at 5 A g^−1^. The higher initial capacity of 2D-HCA can be attributed to the larger SSA. The following discharge capacity of 2D-HCA gradually decreases with cycling. After 1,000 cycles, the discharge capacity fades to 222 mA h g^−1^, which is much lower than that of H-2D-HCA (Figure 4C). As for H-C-OII and C-OII, they discharge capacity respectively are 88 and 78 mA g^−1^ after 1,000 cycles, only about a quarter of that of H-2D-HCA (Figure 4C). The corresponding CE of 2D-HCA, H-C-OII, and C-OII were shown in Supplementary Figure 14, which were worse than that of H-2D-HCA. In addition, the electrochemical performance of H-2D-HCA is better than most of N, S co-doped carbon nanomaterials (summarized in Supplementary Table 1).

In view of above results, benefiting from the unique microstructure, H-2D-HCA delivers highly efficient Li-ion storage performance. We performed first principle calculations to further understand the effect of high-level heteroatom doping on the electrochemical performance of carbon nanomaterials. The influences of dopant types and interlayer distance on Li adsorption energy, electrical conductivity and Li ion diffusion barriers were studied in this work.

To evaluate the stability of Li ions adsorbed on the carbon systems, the adsorption energy was calculated with the followed equation:

(1) $$\begin{array}{r} E_{abs}=E_{2}-E_{1}-E_{Li}, \end{array}$$

**Figure 5:**
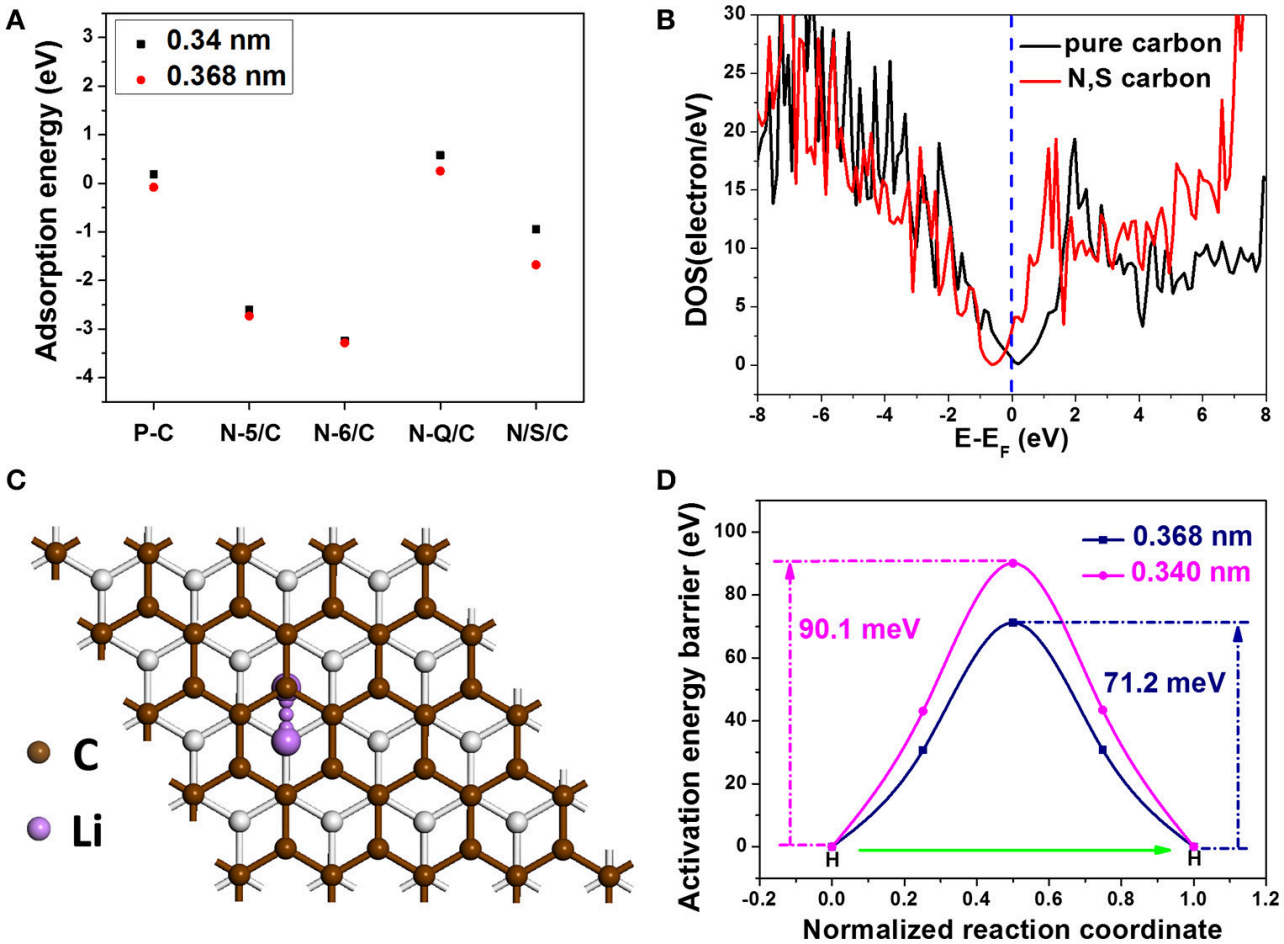
Adsorption energy for the different carbon systems with interlayer spacing of 0.340 and 0.368 nm: P/C, N-6/C, N-5/C, and N/S/C; **(B)** Density of states of the pure carbon and N, S co-doped carbon; **(C)** Illustration of a specific Li ion diffusion path; **(D)** Activation energy barrier curves for the carbon systems with interlayer distance of 0.368 and 0.34 nm.

Where E_abs_ is the adsorption energy, E_2_ is the total energy of the geometry optimized structure with the absorbed Li atoms, E_1_ is the energy of different carbon systems, and E_Li_ is the energy of single Li atom in bulk form (Zhou et al., 2004). Different carbon systems with an expanded interlayer distance of 0.368 nm were considered and their E_abs_ have been calculated: pure carbon (P-C), N-6 doped carbon (N-6/C), N-5 doped carbon (N-5/C), N-Q doped carbon (N-Q/C), and N, S co-doped carbon (N/S/C). For comparison, E_abs_ of above-mentioned carbon systems with an interlayer distance of 0.34 nm were also calculated. In the 0.368 nm spacing case, E_abs_ for the P/C, N-5/C, N-6/C, N-Q/C and N/S/C are −0.08, −2.74, −3.28, 0.58, and −1.68 eV, respectively (the detailed structures are presented in Supplementary Figure 15). Whereas in the 0.34 nm spacing case, the corresponding E_abs_ are 0.18, −2.61, −3.25, 0.25 and −0.94 eV, respectively (Figure 5A). As is known, the more negative E_abs_ value means more stable Li absorption (Ma et al., 2012). Obviously, the carbon system with 0.368 nm interlayer distance have enhanced Li absorption stability. In addition, N-6 and N-5 doped carbon systems have lower E_abs_. This indicates that increasing doping concentration of N-5 and N-6 can obviously improve Li-ion storage ability of carbon nanomaterials. Furthermore, the carbon systems with co-doping of N and S are also have lower E_abs_ compared with pure carbon, indicating more stable Li adsorption.

Another factor that affects Li-ion storage performance is the electrical conductivity of nanocarbon electrodes. In general, materials conductivity is determined by their density of states (DOS) at the Fermi level. We calculated the DOS of carbon systems of pure one and N, S co-doped one (structures can be seen in Supplementary Figures 15, 16). Comparing with the pure carbon system, the N, S co-doped carbon system has higher DOS value (3.0 eV) than the pure carbon (0.63 eV) at Fermi level (Figure 5B), demonstrating its better electric conductivity. The diffusion of kinetics of Li ions also plays a dominant part in the electrochemical performance of nanocarbon anodes. The diffusion kinetics are examined by investigating Li ion diffusion barriers (activation energy). With the climbing-image nudged elastic band (CI-NEB) method, we studied a specific diffusion path, in which Li ions migrate between two adjacent hollow sites (Figure 5C). Convergence tests show that five intermediate images are adequate to accurately describe the activation energy barriers. The calculated activation energy barriers for 0.34 and 0.368 nm are 90.1 and 71.2 m eV, respectively (Figure 5D). This suggests that Li ions can diffusion much easier in carbon nanomaterials with extended interlayer distance. Theoretical calculations indicate that the expanded interlayer distance and the doped high-level N and S heteroatoms in the carbon framework can effectively enhance the Li absorption stability, Li diffusion mobility and electronic conductivity, which facilitates the transportation of Li ions and electrons, and thus improve Li-ion storage performance of H-2D-HCA.

## Conclusions

In summary, by using template-assistant methods, we have developed high-level heteroatom doped two-dimensional carbon architectures (H-2D-HCA) for highly efficient Li-ion storage. The hierarchical structure can alleviate the electrochemical active surface loss, and the porous structure provided rapid diffusion channels for Li ions. More importantly, the highly concentrated heteroatoms of 0.9% sulfur and 15.5% nitrogen are able to created abundant electrochemical active sties to storage Li ions. Also the increased N doping concentration improved the ICE of carbon nanomaterials. Furthermore, the expanded interlayer distance can promote insertion-extraction speed of Li ions. First principle calculations confirmed the enhanced Li absorption stability as well as the electronic conductivity by co-doping of N and S heteroatoms and the accumulated diffusion mobility of Li ions owing to expanded interlayer space. Benefiting from these unique microstructure characteristics and high-level heteroatom doping nature, H-2D-HCA exhibited enhanced Li-ion storage performance. Even at a high current density of 5 A g^−1^, it can exhibit a high discharge capacity of 329 mA h g^−1^ after 1,000 cycles. Because of such superior electrochemical performance, H-2D-HCA can be promising electrode candidates for fast charge-discharge Li-ion batteries.

## Author contributions

ZW and YW did the materials preparation and characterization, as well as electrochemical tests. WW did the first principle calculations. BX and Y-BH supervised this work. All the authors discussed the results and wrote the manuscript.

### Conflict of interest statement

The authors declare that the research was conducted in the absence of any commercial or financial relationships that could be construed as a potential conflict of interest.
