# Supplementary material for: High-Level Heteroatom Doped Two-Dimensional Carbon Architectures for Highly Efficient Lithium-Ion Storage
Source: Front Chem. 2018 Apr 5;6:97. doi: 10.3389/fchem.2018.00097 (PMC5900749; doi:10.3389/fchem.2018.00097)
Supplement: Supplementary file 1 [file Presentation1.PDF]

Supplementary Material

High-Level Heteroatom Doped Two-Dimensional Carbon Architectures for Highly Efficient Lithium-Ion Storage

Zhijie Wang,[1,2,#] Yanyan Wang,[2,#] Wenhui Wang,[3,#] Xiaoliang Yu,[4] Wei Lv,[2] Bin Xiang,[1,*] Yan-Bing He[2,*]

[1]Department of Materials Science & Engineering, CAS Key Lab of Materials for Energy Conversion, Synergetic Innovation Center of Quantum Information Quantum Physics, University of Science and Technology of China, Hefei, Anhui 230026, China

[2]Engineering Laboratory for the Next Generation Power and Energy Storage Batteries, and Engineering Laboratory for Functionalized Carbon Materials, Graduate School at Shenzhen, Tsinghua University, Shenzhen, Guangdong 518055, China

[3]China Key Laboratory of Optoelectronic Devices and Systems of Ministry of Education and Guangdong Province, College of Optoelectronic Engineering, Shenzhen University, Shenzhen 518060, China

[4]Center for Green Research on Energy and Environment Materials, National Institute for Materials Science, Tsukaba 305-0047, Japan

[#] The authors contributed equally to this work.

*** Correspondence:**
Bin Xiang
binxiang@ustc.edu.cn

Yan-Bing He
he.yanbing@sz.tsinghua.edu.cn

1 Supplementary Figures and Tables

1.1 Supplementary Figures

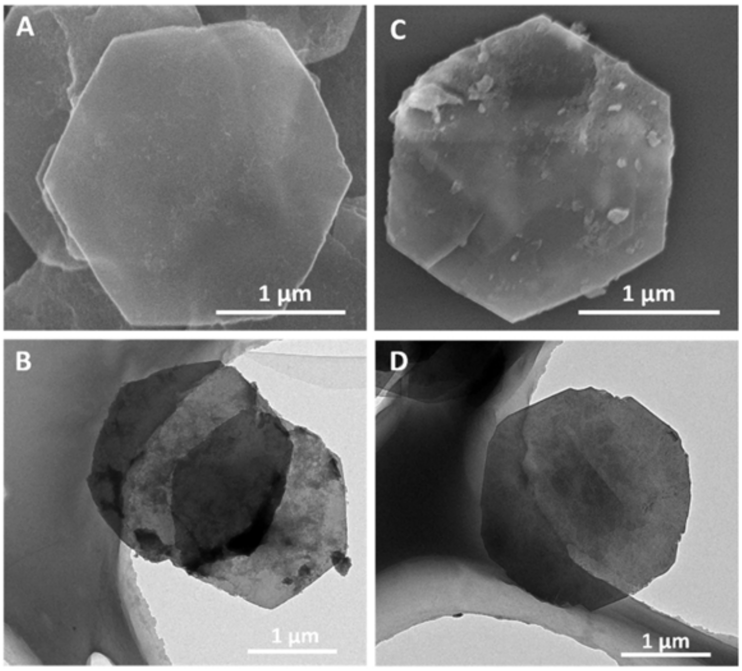

Supplementary Figure 1. (A) SEM image and (B) TEM image of Mg-Al LDH, and (C) SEM image and (D) TEM image of Mg-Al LDO.

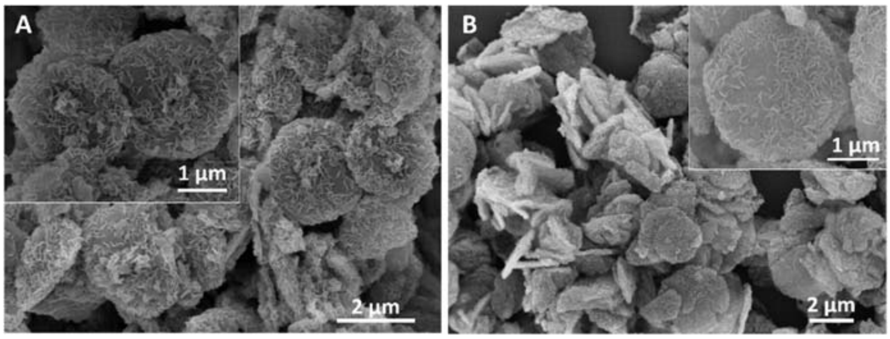

Supplementary Figure 2. SEM image of (A) RLDH-OII and (B) RLDH-OII-M.

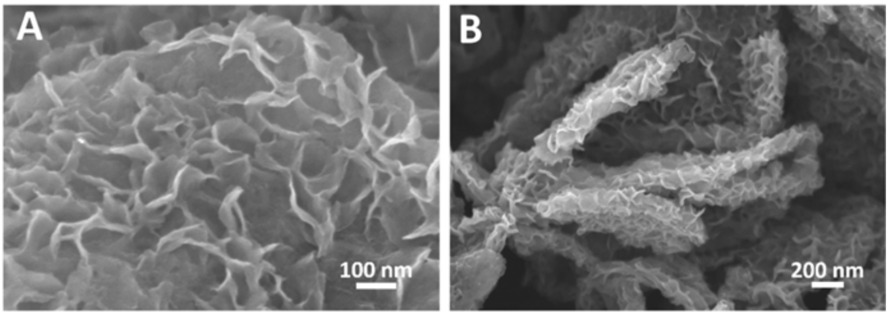

Supplementary Figure 3. (A) High-magnification SEM image and (B) cross-section SEM image of H-2D-HCA.

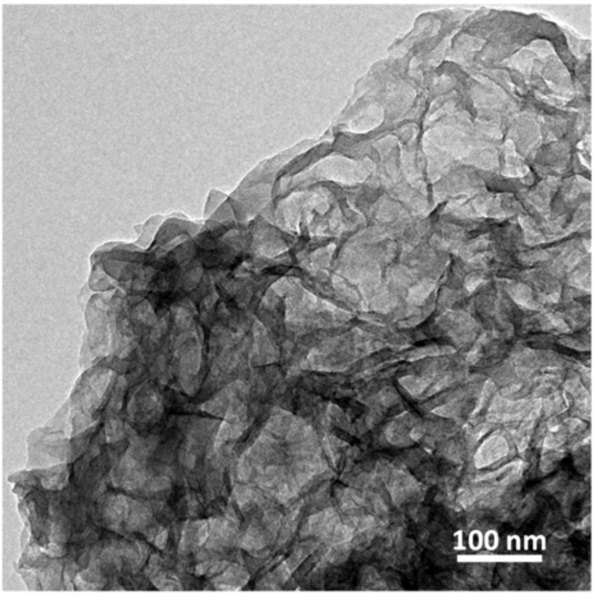

Supplementary Figure 4. TEM image of H-2D-HCA.

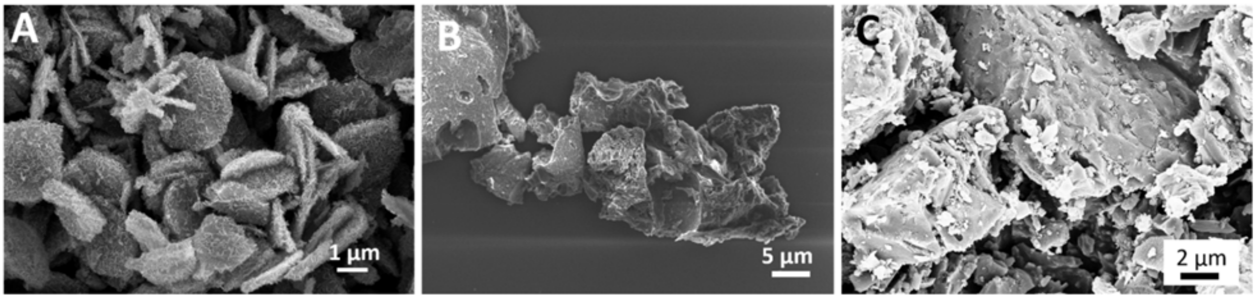

Supplementary Figure 5. SEM image of (A) 2D-HCA, (B) H-C-OII and (C) C-OII.

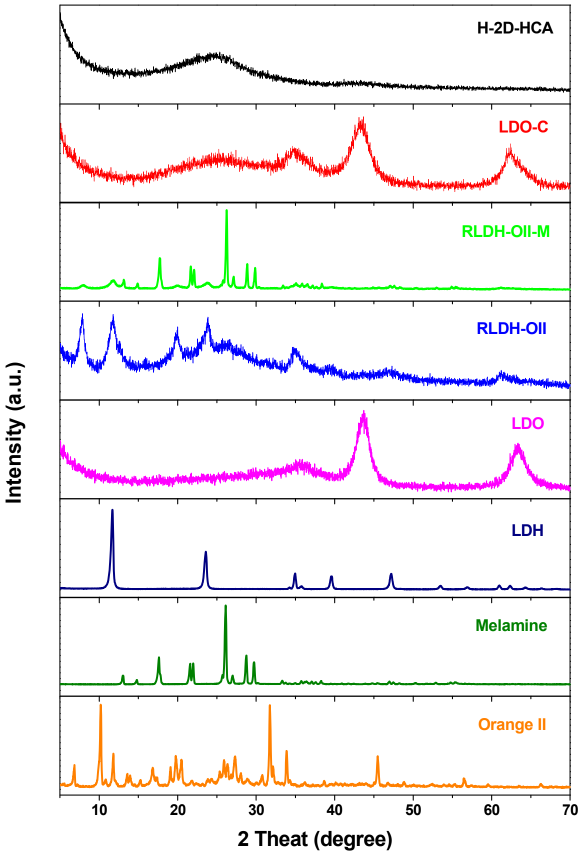

Supplementary Figure 6. XRD patterns of OII, melamine, LDH, DLO, RLDH-OII, RLDH-OII-M, LDO-C and H-2D-HCA. It can be easily seen that after OII adsorption process, LDO was rehydrated to LDH, and after carbonization process, RLDH was calcined to LDO again. In addition, the presences of OII in RLDH-OII and melamine in RLDH-OII-M were also verified.

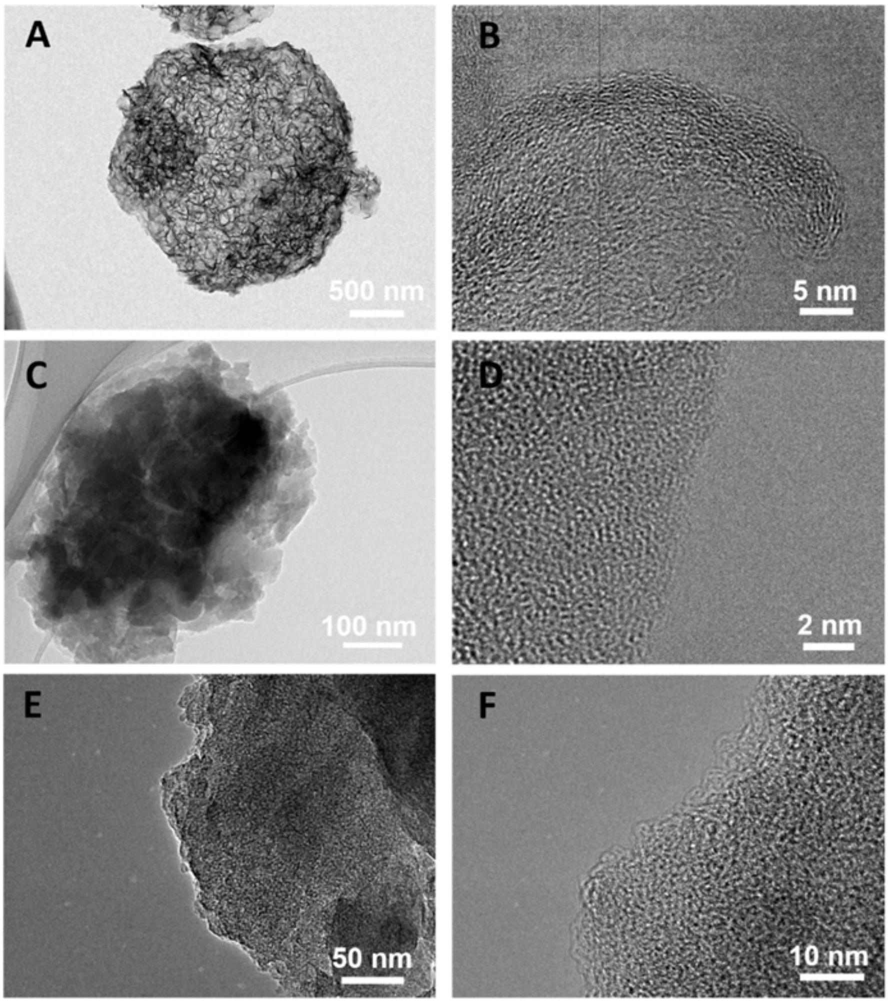

Supplementary Figure 7. (A) SEM image and (B) HRTEM image of 2D-HCA; (C) SEM image and and (D) HRTEM image of H-C-OII; (E) SEM image and (F) and HRTEM image of C-OII.

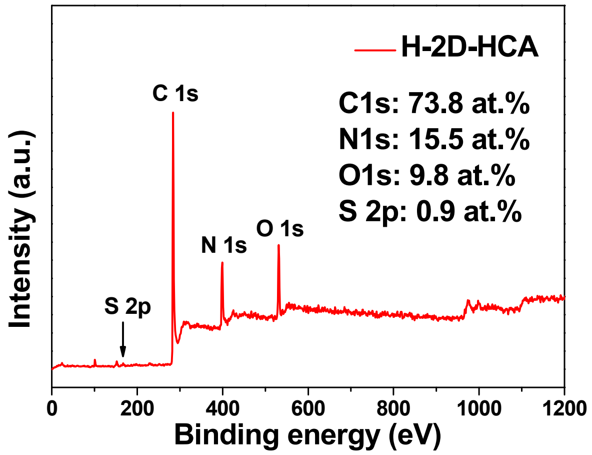

Supplementary Figure 8. XPS spectrum of H-2D-HCA.

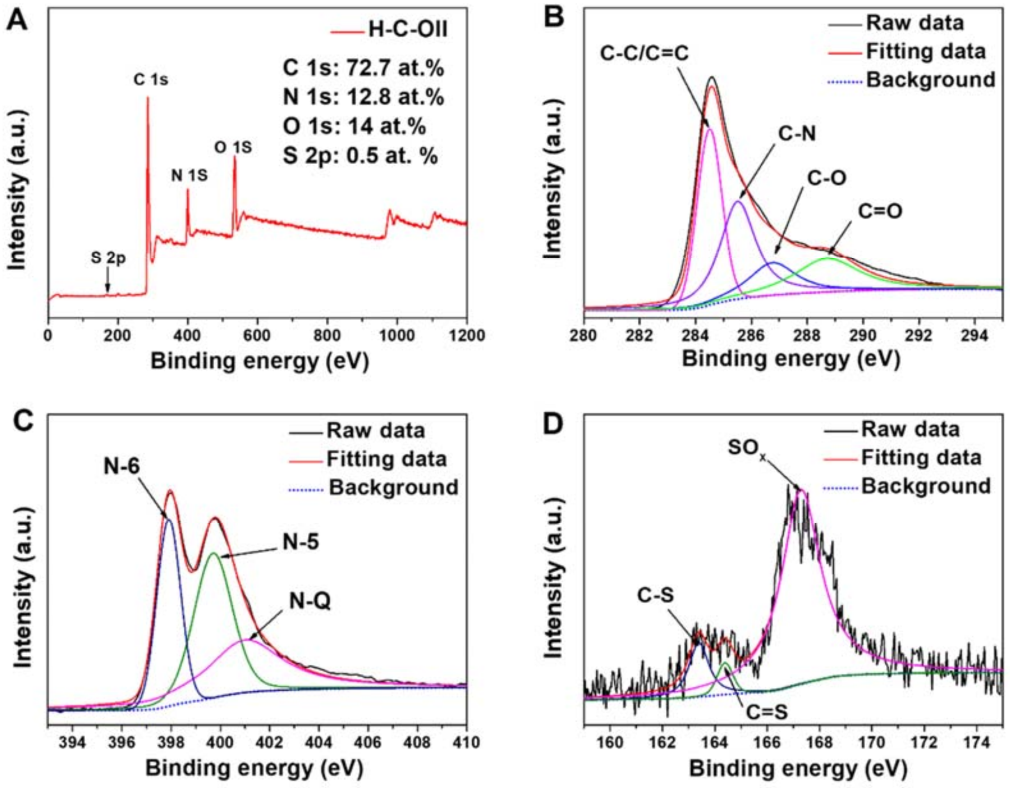

Supplementary Figure 9. (A) XPS spectrum of H-C-OII; (B) High resolution spectrum of C1s; (C) High-resolution spectrum of N1s; (D) High-resolution of S2p.

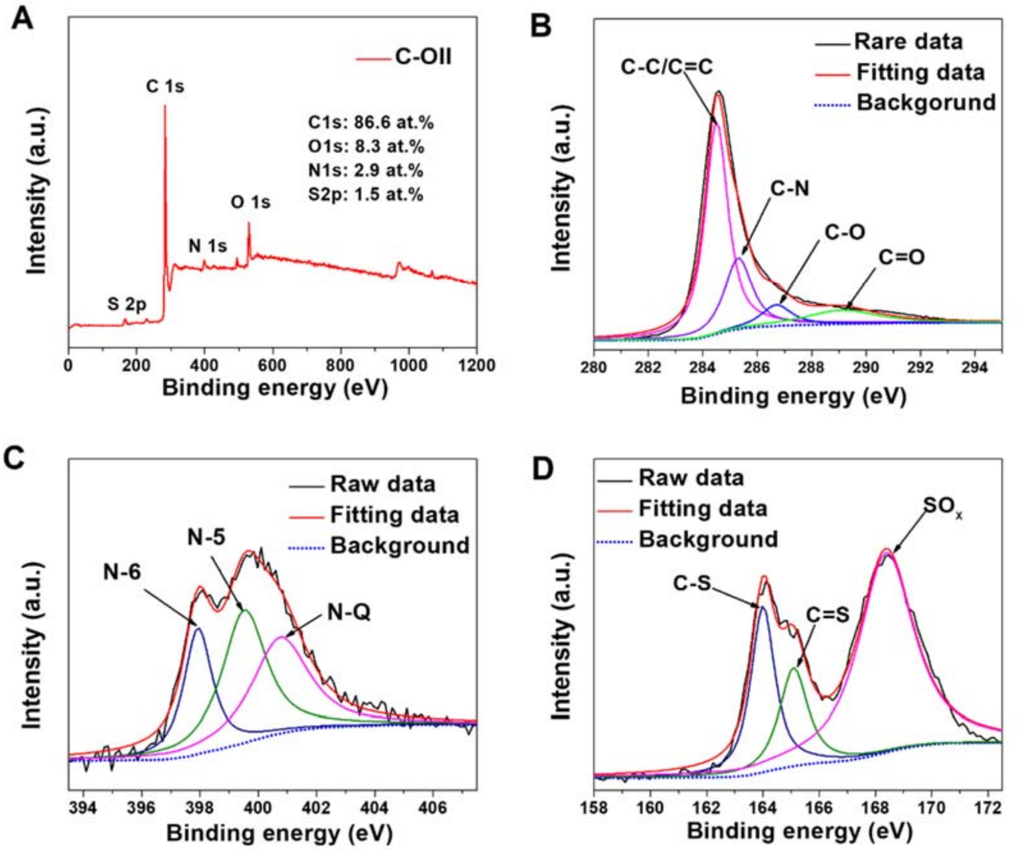

Supplementary Figure 10. (A) XPS spectrum of C-OII; (B) High resolution spectrum of C1s; (C) High-resolution spectrum of N1s; (D) High-resolution of S2p.

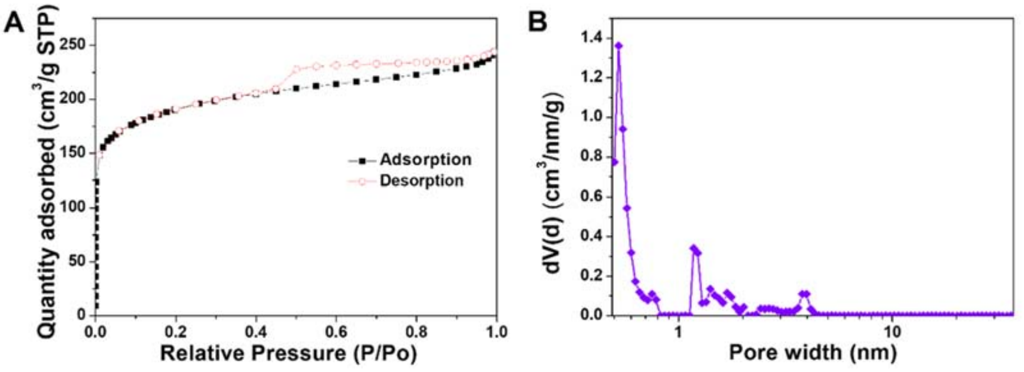

Supplementary Figure 11. (A) N_2 adsorption/desorption isotherms and (B) Pore-size distribution curve and inset: enlarged pore-size distribution of H-C-OII.

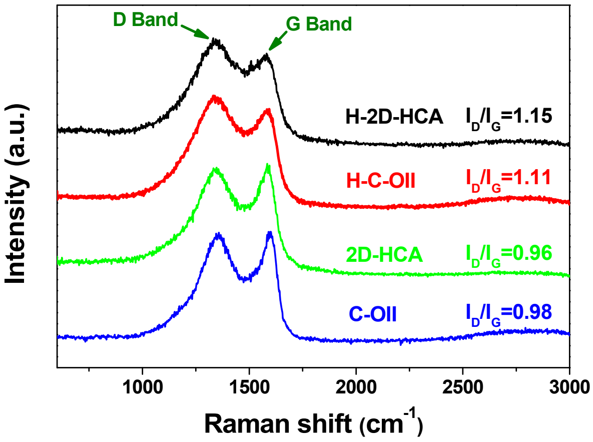

Supplementary Figure 12. Raman spectra of N-2D-HCA, H-C-OII, 2D-HCA and C-OII.

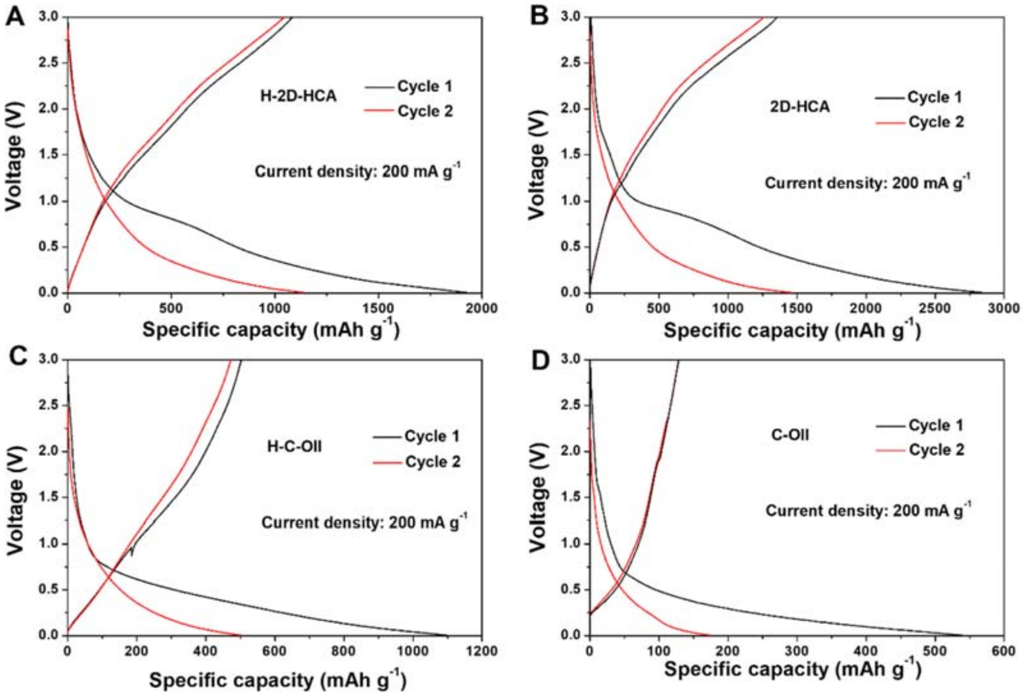

Supplementary Figure 13. Voltage profiles of (A) H-2D-HCA, (B) 2D-HCA, (C) H-C-OII and (D) C-OII for the first two cycles. The current density is 200 mA g^{-1}.

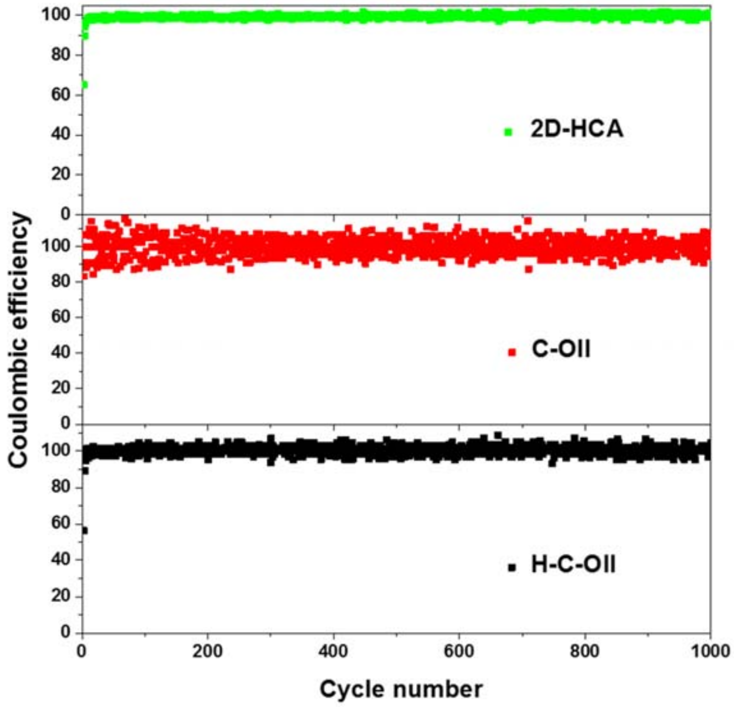

Supplementary Figure 14. Columbic efficiency of 2D-HCA, C-OII and H-C-OII during long-term tests.

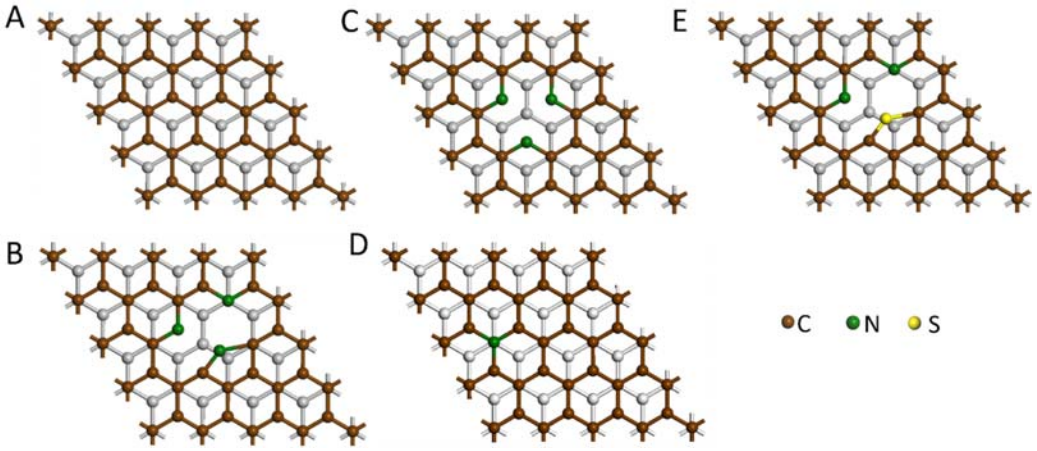

Supplementary Figure 15. Illustration for different carbon systems. (a) P/C; (B) N-5/C; (C) N-6/C; (D) N-Q/C and (E) N/S/C.

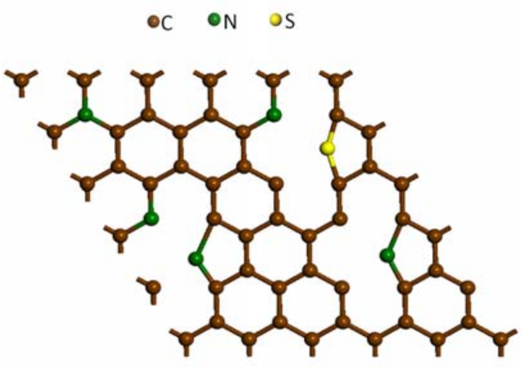

Supplementary Figure 16. Structural illustration of N, S co-doped carbon system for DOS calculation.

1.2 Supplementary Tables

Supplementary Table 1. Summary and comparison about heteroatom doping level and electrochemical performance of different N, S co-doped samples.

Samples	Doping level		Electrochemical performance			Reference
	N (%)	S (%)	Current density (mA g^{-1})	Cycle number	Capacity (mA h g^{-1})	
H-2D-HCA	15.5	0.9	5000	1000	329.2	This work
NSGs	Not present		100	500	490	Zhou et al., 2015
ACSB	2.2	0.97	372	100	261.5	Xu et al., 2015
SNPC	14.82	3.86	100	50	675	Zhuang et al., 2015
NSPCs	3.8	1.05	780	120	1175	Zhang et al., 2016
3D NS-GSs	4.6	1.5	500	80	725	Sun et al., 2015
SNGA	1.15	2.09	100	100	441	Shan et al., 2016
NS-G	1.76	0.86	5000	1400	297	Ai et al., 2014

Supplemental Table S2. Electrochemical performance of different samples for the initial cycle at current density of 200 mA g^{-1}.

Samples	Discharge capacity (mA h g^{-1})	Charge capacity (mA h g^{-1})	Efficiency (%)
H-2D-HCA	1924	1084	56.4
	1136	1044	91.8
2D-HCA	2834	1356	47.8
	1453	1257	86.5
H-C-OII	1098	502	45.7
	502	472	94.1
C-OII	538	128	23.8
	173	128	73.5
